# Treatment of Milk-fever in New Jersey

**Published:** 1898-08

**Authors:** 


If a case of milk-fever could survive the treatment prescribed by a recent writer in the *Jersey Bulletin* it surely would be proof almost against a rifle-bullet. If pregnancy in cows is to be surrounded with such preparations there will soon be a new occupation for many people, as bovine maternity-nurses, and central headquarters or directories where these may be obtained in all large dairy-districts.

